# Dietary branched-chain amino acids intake, glycemic markers, metabolic profile, and anthropometric features in a community-based sample of overweight and obese adults

**DOI:** 10.1186/s12902-023-01459-3

**Published:** 2023-09-25

**Authors:** Ensiye Soleimani, Fariborz Rashnoo, Mahdieh Abbasalizad Farhangi, Babak Hosseini, Faria Jafarzadeh, Amir Shakarami, Yoones Sadabadi

**Affiliations:** 1 Tabriz Health Services Management Research Center, Tabriz University of Medical Sciences, Tabriz, Iran; 2 Department of General and Minimally Invasive surgery, Loghman Hakim Hospital, Shahid Beheshti University of Medical Sciences, Tehran, Iran; 3 Department of Surgery, School of Medicine, Laparoscopy Research Center, Shiraz University of Medical Sciences, Shiraz, Iran; 4 Assistant Professor of Endocrinology & Metabolism, Department of Internal Medicine, School of Medicine, North Khorasan University of Medical Sciences, Bojnourd, Iran; 5 Department of Cardiovascular Medicine, Assistant Professor of Cardiology, Lorestan University of Medical Sciences, Khorramabad, Iran; 6 Faculty of Dentistry, Shahed University, Tehran, Iran

**Keywords:** BCAA, Metabolic and glycemic markers, Anthropometric measurements, LDL

## Abstract

**Background:**

Existing research provides conflicting evidence regarding the relationship between estimated branched-chain amino acid (BCAA) intake and metabolic, glycemic markers, and anthropometric characteristics. This research seeks to examine the association between estimated dietary BCAA consumption and glycemic, and metabolic markers, as well as anthropometric parameters in adults classified as overweight or obese.

**Methods:**

In this cross-sectional analysis, we gathered data from 465 overweight and obese individuals aged between 18 and 37 years. To evaluate dietary data, we employed the food frequency questionnaire, and the BCAA content in foods was determined via the United States Department of Agriculture website. We utilized ELISA kits to measure fasting blood glucose (FBS) and lipid profile markers, and additionally calculated low-density lipoprotein (LDL) and insulin sensitivity markers. We assessed sociodemographic status, physical activity (PA), and anthropometric attributes through a method recognized as both valid and reliable. For statistical analysis, we conducted analyses of covariance (ANCOVA), making adjustments for variables including sex, PA, age, energy, and body mass index (BMI).

**Results:**

Upon adjusting for confounders, those in the highest tertiles of BCAA intake exhibited an increase in weight, BMI, waist circumference (WC), waist-to-hip ratio (WHR), and fat-free mass (FFM). Conversely, they demonstrated reduced fat mass (FM) (%) and FM (kg) compared to their counterparts in the lowest tertiles (P < 0.05). Additionally, there was a noted association between greater estimated BCAA intake and reduced LDL levels. Nonetheless, our findings did not reveal a significant relationship between dietary BCAA and glycemic indices.

**Conclusions:**

From our findings, an increased estimated intake of BCAA seems to correlate with diminished serum LDL concentrations. To gain a more comprehensive understanding of this association, it is imperative that further experimental and longitudinal studies be conducted.

## Background

In the past few decades, metabolic diseases have emerged as some of the most significant public health problems worldwide. Obesity, fatty liver, and diabetes are now among the most prevalent metabolic diseases across all age groups. The obesity epidemic has been recognized by the World Health Organization (WHO) as one of the top 10 global health problems [1–5]. Obesity has reached epidemic proportions in many countries around the world and is closely associated with several chronic diseases, including metabolic disease. Branched-chain amino acids (BCAAs) are essential amino acids with nonlinear aliphatic side chains, including isoleucine, leucine, and valine [6, 7]. A diet rich in BCAAs has been associated with metabolic health, including body weight regulation, muscle protein synthesis, and glucose and lipid homeostasis [8–10]. However, some other studies show different results; according to new research, reducing the amount of BCAAs in the diet may reverse the trend of diet-induced obesity [11–15].

Studies indicate that lifestyle modifications, such as weight loss, increased physical activity (PA), and dietary changes, can enhance glucose tolerance and improve lipid profiles [16–23]. In general, BCAA plays several important metabolic and physiological roles, beyond being considered substrates for the synthesis of proteins [24]. Reports show that BCAA serves as signaling molecules that regulate the metabolism of glucose, lipid, and protein [24]. An increase in the plasma level of BCAA is likely associated with insulin sensitivity [25]. In a study conducted on women (mean age = 54.9 years, SD = 7.2 years), there was an inverse relationship between increased serum levels of BCAAs and lipid abnormalities [26]. Yet, prior studies investigating the relationship between BCAA and genetics concerning diabetes and insulin resistance have shown conflicting results [27–30]. Also, it remains debatable whether the dietary intake of these amino acids is associated with insulin resistance and dyslipidemia [25, 26].

The nutritional status of adults is assessed through several methods, with anthropometric measurements being the most well-known [31, 32]. Obesity leads to changes in body composition, which in turn affect energy expenditure, diet, fat-free mass, and fat mass [33–35]. In mice, an increased intake of protein and BCAAs has been linked to both elevated circulating BCAA levels and changes in body composition [36–39]. However, no study to date has examined the correlation between the estimated intake of BCAA and both metabolic and glycemic markers and anthropometric indicators in overweight and obese adults.

## Methods

### Participant population

**Fig. 1:**
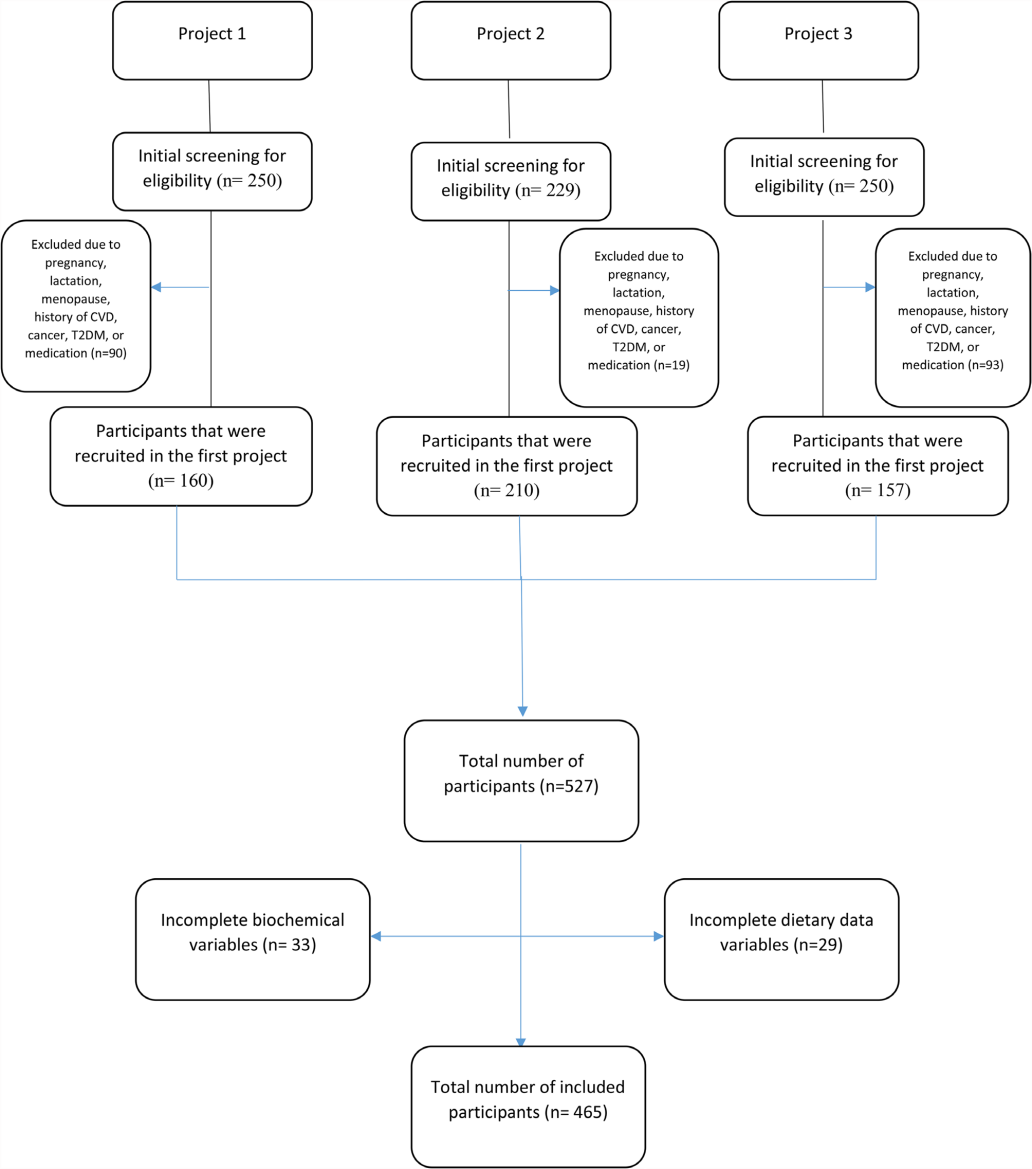
Study flowchart

This cross-sectional study comprised 465 overweight and obese individuals from Tabriz, Iran. Furthermore, the study only included subjects who were in good general health. The participants were selected from three recent projects conducted at the Tabriz University of Medical Sciences [40–42]. The study flowchart is illustrated in (Fig. 1). HOMA-IR was used to calculate sample size [43] z = 1.96, σ = 4.41, δ = 0.27; Using the formula: n=$\frac{{Z}^{2}\times {\sigma }^{2}}{{\delta }^{2}}$; the total sample count reached 465, accounting for 10% missing data. The sample size was determined with α = 0.05 and β = 0.2, yielding power of 80%. For categorization, given the 80% power, dividing the data into tertiles was deemed the optimal approach, both to avoid false positives from multiple comparisons and false negatives due to insufficient power [44, 45].

Inclusion criteria included: consent to participate in the study, a BMI greater than 25 kg/${m}^{2}$, and age between 18 and 37 years. Exclusion criteria included: a history of hypertension, PCOS, CVD, diabetes mellitus or prediabetes, impaired liver or renal function, bariatric surgery, regular use of medications such as oral contraceptive pills, weight-altering medications, BCAA, and high-protein supplements, smoking, alcohol and drug use, pregnancy, currently breastfeeding, and menopause. All participants completed and signed a written informed consent form. The ethics committee of the Tabriz College of Medical Sciences approved the study proposal (Code: IR.TBZMED.REC.1398.460).

### Dietary BCAAs calculation

Dietary intake was assessed using a 168-item food frequency questionnaire (FFQ) [46, 47]. The Nutritionist 4 software (version 7.0; N Squared Computing, Salem, OR) was employed to calculate calories and nutrients in the Iranian diet. The USDA database was applied to determine the BCAAs content of each food product. By entering the name of each product into the USDA website [48], the amount of BCAAs per 100 g of each food was calculated, divided by 100, and then multiplied by the gram of the same food. Finally, the total amount of all BCAAs in the different foods was added to obtain the total amount of BCAAs.

### Sociodemographic, anthropometric, and physical activity measurements

A nutritionist recorded socio-demographic data, including age, sex, and education level, as well as anthropometric measures like body mass index (BMI), height, weight, waist circumference (WC), hip circumference (HC), and waist-to-hip ratio (WHR). A Seca 753E electronic scale was used to measure the weight of the subjects with minimal deviation (accurate to 0.1 kg). The BMI of the subjects was determined using the formula (kg/$${m}^{2})$$, and their standing height was measured (to the nearest 0.1 cm) without shoes. The WHR was derived by dividing the WC by the maximum HC for each participant. After 8 h of fasting, the body composition of participants was assessed using a bioelectrical impedance analysis (BIA) device (Tanita, BC-418 MA, Tokyo, Japan) while they were dressed in light clothing. Though two to three hours of fasting is typically sufficient to measure body composition, participants in this study were not restricted from drinking water. Other criteria for body composition measurement that we adhered to in our study included: no smoking, alcohol, or caffeine intake, no consumption of certain spices such as black pepper, mustard, paprika, and red hot chili peppers 24 h before measurement, refraining from exercise 4 to 6 h prior to measurement, no jewelry or cell phone use during the body composition analysis, wearing no heavy clothing, and avoiding food and water consumption at least 2 h before measurement [49]. The short form of the International Physical Activity Questionnaire (IPAQ), which consists of seven simple questions, was used to assess the level of PA. Its validity and reliability have been previously established [50].

### Biochemical measurements

All participants underwent a blood draw from the brachial vein after an 8-hour fasting period. Serum samples were stored for laboratory analysis by being frozen at -86 °C. Fasting blood glucose (FBS), fasting insulin (FI), total cholesterol (TC), triglycerides (TG), and high-density lipoprotein (HDL) were determined using an ELISA (enzyme-linked immunosorbent assay) kit, following the manufacturer’s instructions. The low-density lipoprotein (LDL) concentration was determined using the Friedewald Eq. (51). The homeostatic model of insulin resistance (HOMA-IR) was assessed using the formula fasting insulin (µIU/mL) × fasting blood glucose (mmol/L)/22.5, and quantitative insulin sensitivity index (QUICKI) as 1/[log fasting insulin (mU/L) + log (fasting plasma glucose (mmol/L) ×18.0182)] [52, 53].

### Statistical analysis

The data gathered were analyzed using SPSS software (version 21.0; SPSS Inc, Chicago, IL). A P-value of less than 0.05 was considered significant. Qualitative data were represented as numbers and percentages (%), while quantitative data were expressed as mean and standard deviation (SD). The Shapiro-Wilk test (p > 0.05) was used to confirm normality, and Levene’s test (p > 0.05) verified the equality of error variances. Bonferroni’s post hoc multiple comparison analysis indicated significant mean differences between groups. To compare the biochemical and anthropometric variables, we used analysis of covariance (ANCOVA), the general linear model (GLM), and univariate analysis, with adjustments for confounding factors such as age, sex, BMI, PA, and energy intake.

## Results

A total of 527 men and women, aged between 18 and 37 years, participated in this study, with 465 subjects completing the measurements. Table 1 presents the general characteristics, anthropometric data, and body composition measurements of the participants across the tertiles of BCAA. A significant difference was observed in weight, height, BMI, WC, HC, WHR, FM, and FFM across the BCAA categories. Furthermore, after adjustments for age, sex, calorie intake, and physical activity, participants in the highest tertiles of BCAA presented higher weight (P = 0.005), BMI (P = 0.007), WC (P = 0.002), WHR (P = 0.017), FFM (%) (P = 0.041), and FFM (kg) (P = 0.035) compared to those in the lowest tertiles. Conversely, after adjusting for age, sex, calorie intake, and physical activity, subjects in the lowest tertiles of BCAA exhibited higher FM (%) (P = 0.005) and FM (kg) (P = 0.038) compared to those in the highest.

**Table 1:** General characteristics and anthropometric measurements of study participants across different tertiles of dietary intake of BCAA

Variables	BCAA				
	T1 (n = 155) (6.99–16.32)	T2 (n = 155) (16.35–23.77)	T3 (n = 155) (23.87–93.87)	*P	**P
**Age (year)**	35.67(11.76)	36.74(10.75)	36.61(10.96)	0.652	0.824
**Gender (male %)**	62(40.0)	94(61.0)	108(69.2)	0.000	-
**Marital status (single %)**	116(74.8)	89(57.8)	102(65.4)	0.080	-
**Education (university graduate %)**	52(33.5)	50(32.5)	58(37.2)	0.500	-
**Occupation status (student %)**	112(72.3)	114(74.0)	122(78.2)	0.227	-
**Weight (kg)**	80.25(17.63)	89.61(17.21)	89.25(15.61)	0.000	**0.005†**
**Height (cm)**	166.57(9.28)	169.61(10.27)	170.84(9.75)	0.000	0.952
**BMI (kg/m2)**	28.97(6.18)	31.20(6.04)	30.69(5.56)	0.003	**0.007†**
**WC (cm)**	94.55(15.71)	102.39(15.08)	101.36(15.09)	0.000	**0.002†**
**HC (cm)**	109.66(10.81)	112.68(11.74)	111.41(9.91)	0.050	0.076
**WHR (cm)**	0.85(0.09)	0.90(0.09)	0.90(0.09)	0.000	**0.017†**
**FM (%)**	37.49(14.84)	35.65(13.12)	32.19(11.88)	0.002	**0.005‡‡**
**FM (kg)**	30.66(13.73)	32.04(12.28)	29.00(11.43)	0.102	**0.038‡‡**
**FFM (%)**	69.32(15.39)	66.30(12.43)	69.39(12.03)	0.069	**0.041**
**FFM (kg)**	54.09(10.80)	58.37(12.01)	61.22(12.14)	0.000	**0.035‡**
**PA (met-hour/week)**	3.08(2.90)	3.11(4.26)	3.85(5.99)	0.238	0.656

BMI: body mass index; WC: waist circumference; HC: hip circumference; WHR: waist-to-hip ratio; FM: fat mass; FFM: fat free mass; PA: physical activity. Data are presented as mean ± SD or percent; *Obtained from the one-way analysis of variance (ANOVA) or Chi-squared tests, where appropriate; P Significant at P < 0.05; 95th confidence intervals of the difference in parentheses **Obtained from ANCOVA model after adjustment for the confounding effects of age, sex, calorie intake and physical activity; P Significant at P < 0.05; 95th confidence intervals of the difference in parentheses

† post hoc Tukey signature difference between 1st tertile and 2nd tertile

‡ post hoc Tukey signature difference between 1st tertile and 3rd tertile

‡‡ post hoc Tukey signature difference between 2nd tertile and 3rd tertile

Table 2 presents the estimated intake of BCAA for participants across the tertiles of BCAA. An upward trend was observed in energy, protein, CHO, cholesterol, zinc, phosphorus, calcium, manganese, fluoride, vitamin C, vitamin B1, vitamin B2, vitamin B3, vitamin B9, vitamin B12, and vitamin D across BCAA tertiles (from T1 to T3). This trend was evident in both the crude analysis and after adjustments for age, sex, BMI, physical activity, and calorie intake (P ≤ 0.05).

**Table 2:** Energy adjusted dietary intakes of study participants across different tertiles of dietary intake of BCAA

Variables	BCAA				
	T1 (n = 155) (6.99–16.32)	T2 (n = 155) (16.35–23.77)	T3 (n = 155) (23.87–93.87)	*P	**P
**Energy (kcal)**	2107.56(639.66)	2886.33(830.09)	3729.59(1212.15)	0 < 0.001	0 < 0.001†‡a
**Branched Chains Amino Acids (gr/day)**	12.95(2.13)	19.93(2.26)	33.63(11.35)	0 < 0.001	0 < 0.001†‡a
**Protein (g/day)**	68.23(18.60)	92.69(21.84)	132.63(49.51)	0 < 0.001	0 < 0.001‡a
**Fat (g/day)**	69.26(25.45)	95.24(40.91)	127.54 (57.98)	0 < 0.001	0.540
**CHO (g/day)**	320.29(119.31)	437.22(137.50)	536.03(181.35)	0 < 0.001	0.006a
**Total Fiber (g/day)**	34.08(21.04)	54.28(29.91)	71.62(51.33)	0 < 0.001	0.499
**SFA (g/day)**	20.66(8.15)	27.75(12.32)	38.49(21.93)	0 < 0.001	0.476
**MUFA (g/day)**	22.41(8.78)	31.41(15.30)	42.14(20.51)	0 < 0.001	0.834
**PUFA(g/day)**	15.63(6.70)	22.08(13.67)	27.74(15.05)	0 < 0.001	0.609
**Cholesterol (mg/day)**	179.38(103.18)	263.13(217.67)	394.90(231.99)	0 < 0.001	0 < 0.001‡a
**Sodium (mg/day)**	3152.19(1384.36)	4362.41(2161.64)	5289.44(2507.03)	0 < 0.001	0.171
**Iron (mg/day)**	17.28(6.17)	23.92(13.53)	29.73(11.31)	0 < 0.001	0.195
**Magnesium (mg/day)**	345.25(137.22)	479.16(145.96)	636.23(285.89)	0 < 0.001	0.071
**Zinc (mg/day)**	9.49(3.38)	12.89(4.03)	18.28(8.81)	0 < 0.001	0.006a
**Phosphorus (mg/day)**	1206.98(387.55)	1657.10(501.58)	2197.03(792.94)	0 < 0.001	0 < 0.001‡a
**Calcium (mg/day)**	910.68(305.89)	1234.33(410.10)	1636.01(700.41)	0 < 0.001	0.004‡a
**Potassium (mg/day)**	3335.10(1566.32)	4451.69(1600.62)	5608.71(2248.20)	0 < 0.001	0.904
**Copper (mg/day)**	1.97(0.70)	2.51(0.93)	3.48(2.00)	0 < 0.001	0.321
**Manganese (mg/day)**	4.95(2.36)	7.54(2.88)	10.26(4.76)	0 < 0.001	0 < 0.001†‡a
**Selenium (mg/day)**	75.52(63.93)	116.21(71.39)	146.27(99.50)	0 < 0.001	0.218
**Fluoride (mg/day)**	3029.03(3542.05)	4108.20(3895.14)	7788.42(7265.19)	0 < 0.001	0 < 0.001†‡a
**Chromium (mg/day)**	0.09(0.07)	0.14(0.09)	0.16(0.13)	0 < 0.001	0.293
**Vitamin C (mg/day)**	190.25(168.13)	230.10(175.24)	235.91(166.03)	0.037	0 < 0.001‡a
**VitaminB1 (mg/day)**	1.72(0.56)	2.43(0.73)	3.19(1.21)	0 < 0.001	0.008
**VitaminB2 (mg/day)**	1.63(0.58)	2.31(0.74)	3.21(1.31)	0 < 0.001	0 < 0.001
**VitaminB3 (mg/day)**	19.77(6.05)	27.71(7.15)	37.28(13.52)	0 < 0.001	0 < 0.001
**VitaminB6 (mg/day)**	1.54(0.66)	2.16(0.88)	2.63(1.03)	0 < 0.001	0.399
**VitaminB9 (µg/day)**	419.86(205.97)	610.22(231.46)	803.51(378.49)	0 < 0.001	0.015‡a
**VitaminB12 (µg/day)**	3.54(1.97)	4.86(2.97)	9.61(15.06)	0 < 0.001	0.078
**VitaminB5 (mg/day)**	5.15(1.80)	6.66(2.43)	8.44(3.34)	0 < 0.001	0.926
**VitaminB8 (mg/day)**	27.44(15.45)	36.83(15.60)	44.34(21.17)	0 < 0.001	0.852
**Vitamin A (RAE/day)**	735.05(448.10)	945.26(629.34)	1422.96(1480.44)	0 < 0.001	0.491
**Vitamin D (µg/day)**	1.52(1.07)	2.03(1.48)	2.62(2.22)	0 < 0.001	0.003‡
**Vitamin K (µg/day)**	180.17(136.27)	223.39(147.38)	327.92(292.63)	0 < 0.001	0.071
**Vitamin E (mg/day)**	9.02(6.34)	13.90(9.47)	15.45(9.53)	0 < 0.001	0.198

CHO: Carbohydrate; SFA: Saturated fatty acids; MUFA: Monounsaturated fatty acids; PUFA: Polyunsaturated fatty acids *P Significant at P < 0.05; 95th confidence intervals of the difference in parentheses. **P values are obtained from ANCOVA model after adjustment for the confounding effects of age, sex, BMI and physical activity, calorie intake

† post hoc Tukey signature difference between 1st tertile and 2nd tertile

‡ post hoc Tukey signature difference between 1st tertile and 3rd tertile

a: post hoc Tukey signature difference between 2nd tertile and 3rd tertile

Table 3 displays the distribution of food groups across the tertiles of BCAA. Significant differences emerged in the consumption of fruits, vegetables, MFP (Meat, Fish, Poultry), dairy, grains, nuts, beans, and fiber across the BCAA tertiles. Furthermore, in the adjusted model, the tertiles of BCAA showed a statistically significant association with fruits, MFP, dairy, and grains.

**Table 3:** Consumption rates of 8 diet components (grams/day)

Variables	BCAA				
	T1 (n = 155) (6.99–16.32)	T2 (n = 155) (16.35–23.77)	T3 (n = 155) (23.87–93.87)	*P	**P
**Fruits (g/d)**	509.77(502.51)	654.96(579.24)	622.02(542.65)	0.048	0 < 0.001†
**Vegetables (g/d)**	272.45(191.68)	325.53(185.47)	396.94(316.18)	0 < 0.001	0.960
**MFP (g/d)**	57.09 (31.70)	73.34(40.51)	127.51(132.40)	0 < 0.001	0 < 0.001††
**Dairy (g/d)**	253.13(170.32)	330.23(240.01)	462.28(394.62)	0 < 0.001	0.009††
**Grains (g/d)**	409.36(155.69)	563.23(206.41)	731.59(289.61)	0 < 0.001	0 < 0.001††
**Nuts(g/day)**	11.52(15.37)	20.10(28.85)	44.68(120.08)	0 < 0.001	0.269
**Beans(g/day)**	33.41(25.35)	47.48(39.21)	66.95(73.48)	0 < 0.001	0.339
**Fiber (g/day)**	13.63(10.22)	18.49(11.08)	22.62(19.03)	0 < 0.001	0.10

MFP; meat, Fish, Poultry. All data are expressed as mean (± SD). P-values derived from one-way ANOVA. *All variables were adjusted for demographic characteristics including age, sex, physical activity and calorie intake. The bold values represent statistically significance

† post hoc Tukey signature difference between 1st tertile and 2nd tertile

‡ post hoc Tukey signature difference between 1st tertile and 3rd tertile

†† post hoc Tukey signature difference between 2nd tertile and 3rd tertile

Table 4 outlines the association between the estimated intake of BCAA and biochemical parameters in both crude and adjusted models. A one-way ANOVA analysis examining the relationship between the BCAA tertiles and biochemical parameters indicated that an increased estimated intake of BCAA correlates with reductions in TC (P = 0.008) and LDL (P = 0.002). Even after adjustments for age, sex, BMI, physical activity, and calorie intake, this relationship remained statistically significant for LDL (P = 0.02).

**Table 4:** Biochemical parameters of study participants across different tertiles of dietary intake of BCAA

Variables	BCAA				
	T1 (n = 155) (6.99–16.32)	T2 (n = 155) (16.35–23.77)	T3 (n = 155) (23.87–93.87)	*P	**P
**FBS (mg/dl)**	84.13(14.47)	87.37(16.81)	88.35(24.21)	0.123	0.453
**Insulin (µ IU/ml)**	14.54(14.55)	15.51(18.50)	14.20(14.44)	0.754	0.758
**TC (mg/dl)**	201.06(47.92)	191.48(38.75)	186.32(40.12)	0.008	0.117
**HDL (mg/dl)**	45.09(10.65)	45.58(9.84)	43.90(10.49)	0.339	0.462
**LDL (mg/dl)**	132.22(47.88)	121.92(34.71)	116.37(37.67)	0.002	0.027‡
**TG (mg/dl)**	141.00(96.36)	148.97(91.98)	146.65(78.52)	0.722	0.590
**HOMA-IR**	3.18(3.42)	3.39(3.98)	3.18(3.34)	0.833	0.844
**QUICKI**	0.34(0.04)	0.33(0.03)	0.33(0.03)	0.747	0.766

FBS: fasting blood sugar; TC: total cholesterol; HDL: high-density lipoprotein; LDL: low-density lipoprotein; TG: triglycerides. *P Significant at P < 0.05; 95th confidence intervals of the difference in parentheses. **P values are obtained from ANCOVA model after adjustment for the confounding effects of age, sex, BMI and physical activity, calorie intake and further adjusted for protein intake for Insulin level

† post hoc Tukey signature difference between 1st tertile and 2nd tertile

‡ post hoc Tukey signature difference between 1st tertile and 3rd tertile

†† post hoc Tukey signature difference between 2nd tertile and 3rd tertile

## Discussion

This cross-sectional study explored possible associations between the estimated intake of BCAA and glycemic, metabolic indices, and anthropometric measurements in overweight and obese adults in Iran. The findings from this study suggest that an elevated dietary intake of BCAA associates with an increase in weight, BMI, WC, WHR, and FFM (P < 0.05). Conversely, participants in the highest tertiles of BCAA exhibited lower FM (kg), FM (%), and LDL when compared to subjects in the lowest tertiles (p < 0.05). We observed no significant association between BCAA and glycemic indices (P > 0.05).

Approximately 80% of serum BCAA levels are determined by the consumption of proteins or BCAAs from food or supplements, while the remaining 20% is influenced by their catabolic metabolites [54, 55]. Our findings align with some of the studies’ results regarding the relationship between serum BCAA concentrations and glycemic, metabolic indices, and anthropometric measurements [8, 13, 14, 25, 39, 56, 57]. Several studies have produced mixed findings on the relationship between increased dietary BCAA and glycemic, metabolic, and anthropometric indices. While some research indicates an association with improvements in these indices [58, 59], others suggest the contrary [14, 43, 57, 60].

To the best of our knowledge, no study has explored the relationship between dietary BCAA intake and glycemic, metabolic factors, and anthropometric measurements within the overweight and obese Iranian population. In our research, while glycemic markers trended upward with increased estimated intake of BCAAs, this correlation wasn’t statistically significant. It’s also noteworthy that findings from similar studies offer conflicting results [28, 61]. Zheng et al. observed that an increase in dietary BCAAs corresponded to a 13% elevated risk of diabetes [61]. Another study found that in a Japanese community, a higher estimated intake of BCAA correlated with a reduced risk of diabetes [28]. Several reasons can explain the observed contradictions: (1) Different populations and countries have varying primary dietary sources that contribute to the total BCAA intake. (2) There may be biases in completing the food frequency questionnaire. (3) Various confounding factors are at play. (4) The methodologies for evaluating results differ, such as comparing insulin resistance versus diabetes. Various studies have suggested that an increased dietary intake of BCAA may enhance glucose metabolism, likely through the stimulation of insulin secretion and activation of cell signaling pathways like mTOR (mechanistic Target of Rapamycin) and AMPK (AMP-activated Protein Kinase) [62, 63]. However, the impact of branched-chain amino acid consumption on fasting glucose and insulin levels may be influenced by other factors, including total caloric intake, other dietary components, physical activity, and individual genetics [28, 29, 64]. One possible explanation for this inconsistency might be the interplay between nutritional intake and genetics. Illustratively, a study by Wang et al. posited that dietary BCAAs could amplify the genetic predisposition to an increased risk of type 2 diabetes (T2D) and elevated fasting glucose levels. They further identified that a higher BCAA intake correlated positively with T2D risk in individuals with a high genetic predisposition. Conversely, this association was negative for those with a low genetic predisposition, implying that BCAAs’ influence on T2D risk might be contingent upon an individual’s genetic makeup [27]. Another study indicated that a high consumption of BCAAs correlates with an elevated risk of type 2 diabetes [28, 29].Further research is required to comprehensively understand this beneficial mechanism.

In our investigation, we found that increasing dietary BCAA intake significantly reduced serum LDL levels. However, we didn’t observe a notable correlation between BCAA and total cholesterol, HDL, or TG. Zhang et al. documented that a higher BCAA consumption corresponded to decreased serum cholesterol levels, aligning partially with our findings [29]. Various studies have demonstrated that BCAA can enhance the lipid profile through the activation of the mTOR pathway, increased insulin secretion, and augmented fat metabolism [55, 62, 65]. Conversely, Yang et al.‘s research indicated that a higher intake of BCAA correlated with dyslipidemia [66]. However, to fully comprehend the mechanism behind the dietary intake of BCAA and its association with the lipid profile, further studies are required.

Although in the present study, an increase in dietary BCAAs associated with a rise in weight, BMI, WC, WHR, and FFM. Conversely, the higher tertiles of dietary BCAA were associated with decreases in FM and FM%. It’s worth noting that adipose tissue and the liver might be the primary sites where BCAAs interact with lipid metabolism [67, 68]. In the research by Ribeiro et al., as well as in our own study, an increase in BCAA intake led to weight gain in older mice and males. However, this gain was primarily attributed to a rise in FFM [69]. Conversely, BCAA metabolism also plays a pivotal role in adipocyte differentiation and lipogenesis [70]. There was a positive correlation between plasma BCAA concentrations and markers of visceral adipose tissue (VAT) as well as insulin resistance [71]. The influence of dietary intake of BCAA on body composition varies based on the amount of these amino acids consumed and other dietary components. Increasing the intake of BCAA can stimulate protein synthesis through enhanced insulin secretion, which influences the fat-free mass. Conversely, by boosting fat metabolism, it can also impact the fat mass [33, 62].

Obesity impedes BCAA catabolism by downregulating the expression of genes linked to the branched-chain alpha-keto acid dehydrogenase complex (BCKD) [71]. The expression level of BCKD mRNA in VAT is diminished in obese women with metabolic syndrome compared to obese women without glucose metabolism issues [72]. Levels of BCAAs and their intermediates, including C3- and C5-acylcarnitine, were elevated in obese individuals [73]. There’s a need for another longitudinal study to bridge this knowledge gap. In our analysis, while we considered factors like age, sex, BMI, PA, and calorie intake, we didn’t account for genetic factors and other variables that are either unknown or inadequately measured. Due to the cross-sectional design of our study, establishing causal relationships is challenging. The use of a semiquantitative dietary assessment questionnaire also poses the risk of recall bias given its subjective nature. Nonetheless, a strength of this research is the examination of a comprehensive range of variables.

## Conclusions

In our cross-sectional study of overweight and obese Iranians, an increase in dietary BCAA was significantly associated with an increase in weight (P = 0.005), BMI (P = 0.007), WC (P = 0.002), WHR (P = 0.017), and FFM (P = 0.035) and a decrease in FM % (P = 0.005) and FM (P = 0.038). In addition, increased intake of BCAAs significantly decreased blood LDL levels (P = 0.027).

## Data Availability

(ADM) The datasets used and/or analyzed during the current study are available from the corresponding author upon reasonable request.

## Abbreviations

FFQ: Food frequency questionnaire
PA: Physical activity
BMI: Body mass index
ANOVA: Analysis of variance
ANCOVA: Analysis of covariance
BIA: Bioelectric impedance analysis
MAC: Mid arm circumference
MFP: Meat Fish Poultry
WC: Waist circumference
WHR: Waist to hip ratio
FFA: Free fatty acids
BCAA: Branched-chain amino acids
TC: Total cholesterol
LDL: Low-density lipoprotein
HDL: High-density lipoprotein
TG: Triglyceride
WHO: World Health Organization
HC: Hip circumference
IPAQ: International Physical Activity Questionnaire
FBS: Fasting blood glucose
FI: Fasting insulin
SD: Standard deviation
GLM: general linear model
VAT: visceral adipose tissue
BCKD: branched-chain alpha-keto acid dehydrogenase complex
FM: Fat mass
BMI: Body mass index
